# Myo-Inositol Supplementation in Suckling Rats Protects against Adverse Programming Outcomes on Hypothalamic Structure Caused by Mild Gestational Calorie Restriction, Partially Comparable to Leptin Effects

**DOI:** 10.3390/nu13093257

**Published:** 2021-09-18

**Authors:** Pedro Castillo, Mariona Palou, Zhi Xin Yau-Qiu, Ana M. Rodríguez, Andreu Palou, Catalina Picó

**Affiliations:** 1 Laboratory of Molecular Biology, Nutrition and Biotechnology (Nutrigenomics and Obesity), University of the Balearic Islands, 07122 Palma, Spain; pedro.castillo@uib.es (P.C.); zx.yau@uib.es (Z.X.Y.-Q.); amrodriguez@uib.es (A.M.R.); andreu.palou@uib.es (A.P.); cati.pico@uib.es (C.P.); 2 CIBER de Fisiopatología de la Obesidad y Nutrición (CIBEROBN), 07122 Palma, Spain; 3 Health Research Institute of the Balearic Islands (IdISBa), 07122 Palma, Spain

**Keywords:** fetal undernutrition, myo-inositol, leptin, lactation period, metabolic programming, arcuate nucleus, paraventricular nucleus, PBMC

## Abstract

We studied whether myo-inositol supplementation throughout lactation, alone and combined with leptin, may reverse detrimental effects on hypothalamic structure and function caused by gestational calorie gestation (CR) in rats. Candidate early transcript-based biomarkers of metabolic health in peripheral blood mononuclear cells (PBMC) were also studied. Offspring of dams exposed to 25% gestational CR and supplemented during lactation with physiological doses of leptin (CR-L), myo-inositol (CR-M), the combination (CR-LM), or the vehicle (CR-V) as well as control rats (CON-V) were followed and sacrificed at postnatal day 25. Myo-inositol and the combination increased the number of neurons in arcuate nucleus (ARC) (only in females) and paraventricular nucleus, and myo-inositol (alone) restored the number of αMSH^+^ neurons in ARC. Hypothalamic mRNA levels of *Lepr* in CR-M and *Insr* in CR-M and CR-LM males were higher than in CR-V and CON-V, respectively. In PBMC, increased expression levels of *Lrp11* and *Gls* in CR-V were partially normalized in all supplemented groups (but only in males for *Gls*). Therefore, myo-inositol supplementation throughout lactation, alone and combined with leptin, reverts programmed alterations by fetal undernutrition on hypothalamic structure and gene expression of potential early biomarkers of metabolic health in PBMC, which might be attributed, in part, to increased leptin sensitivity.

## 1. Introduction

Gestation and lactation are critical developmental windows in the programming of later metabolic health [1,2]. Both epidemiological evidences and animal studies have shown that adverse in-utero conditions can predispose the offspring to overweight/obesity and related metabolic alterations, such as insulin resistance, in adulthood [2,3,4,5]. The altered energy homeostasis control triggered by malnutrition during gestation has been associated in animal models to changes in the structure of key hypothalamic nuclei involved in the control of food intake and energy expenditure [6,7,8]. Regarding lactation, it is clearly established that breast milk represents optimal nutrition during this period [9]. Epidemiological studies have reported that breastfeeding compared to infant formula feeding provides numerous benefits to offspring health in the short term but also in the longer term, exerting a protection against the development of obesity and other associated disorders in adulthood [10,11,12]. The specific mechanisms and factors that account for the benefits of breast milk are still uncertain, but specific milk compounds may play a role. In this regard, leptin, which is naturally present in breast milk [13,14] but not in infant formula [15], has been shown to be biologically relevant in metabolic programming and contribute to the protective effects of breastfeeding [16], but other less known factors may also play a role.

The role of leptin ingested during lactation was first established from indirect epidemiological studies in humans showing a negative correlation between breast milk leptin levels and body weight or body weight gain of infants, particularly of non-obese women [16,17]. Intervention studies in animal models have corroborated the essential role of leptin ingested at physiological doses during the suckling period in the prevention of high-fat diet- and age-related metabolic alterations in adulthood [18,19,20]. Moreover, leptin supplementation during lactation has also been shown to prevent most of the harmful programming effects associated to mild/moderate gestational calorie restriction in rats, both in the short and long term [21,22,23]. More specifically, it prevents alterations in the development of hypothalamic circuits involved in body weight control [22]; restores peripheral nervous system structures, including sympathetic innervation in white adipose tissue [23]; and normalizes the gene expression profile in peripheral blood mononuclear cells (PBMC) that is altered in the offspring of gestational calorie restricted dams at an early age, which is indicative of adverse programming effects [24]. Such effects of leptin during the suckling period have been shown to be translated into a healthier phenotype in adulthood, ameliorating the dysmetabolic phenotype associated to undernutrition during gestation, which includes excess fat accumulation, insulin resistance, hypertriglyceridemia, hepatic steatosis, and adipose tissue inflammation [21]. The effects of leptin have been attributed, in part, to the neurotrophic action that it exerts during the suckling period [25], which may be critical for subsequent sensitivity to this hormone.

Myo-inositol has also been suggested as an interesting milk component for its potential role in the neonate development. On the one hand, myo-inositol levels in infant formulas are generally lower than those naturally present in breast milk [26]. On the other hand, this compound has been found at increased levels in the milk of rat dams subjected to mild/moderate calorie restriction during lactation [27], a condition associated with a healthier metabolic profile of offspring both at a juvenile age [27,28] and in adulthood [28,29,30], contrary to the model of CR during gestation. Interestingly, myo-inositol supplementation at physiological doses during the suckling period has been shown to improve metabolic health in male rats, ameliorating the insulin resistance and hypertriglyceridemia programmed by moderate gestational calorie restriction (CR), particularly evident when animals were exposed to a diabetogenic diet in adulthood [31]. It is noteworthy that the lasting effects of myo-inositol supplementation at physiological doses during the suckling period in adult male rats are comparable, to some extent, to those described for leptin [21] since both compounds have been shown to be capable of reversing, at least in part, the postnatal sequelae in the phenotype associated with fetal malnutrition, particularly in terms of insulin resistance. Thus, it was hypothesized that leptin and myo-inositol could exert similar and/or complementary short-term programming effects during the suckling period, allowing the reversal of detrimental outcomes caused by gestational undernutrition. However, the early programming effects of myo-inositol supplementation during the suckling period and whether it may also reverse neuroanatomical defects in hypothalamic structures acquired by adverse in utero conditions have not been determined. Moreover, taking into account that the concentration of myo-inositol in breast milk displays a certain range of variability [32], as also occurs with leptin, whose concentration is related to maternal adiposity [14,17,33], it would be of interest to assess potential interactions when supplementing with both compounds.

Therefore, the aim of this study was to assess the short-term effects of myo-inositol supplementation, alone and in combination with leptin, during the lactation period, on the hypothalamic structure and function in the male and female offspring of rats exposed to moderate calorie restriction during gestation. In addition, expression levels of selected genes in PBMC previously proposed as early biomarkers of metabolic health [24,34,35] were also studied.

## 2. Materials and Methods

### 2.1. Animals and Experimental Design

The study was performed in 25-day-old male and female Wistar rats from 19 different litters. Animals were housed under controlled conditions (22 °C and 12 h of light-dark cycle) and with free access to tap water and standard chow diet (3.3 kcal·g^−1^; Panlab, Barcelona, Spain) unless otherwise specified.

Briefly, virgin female rats weighing between 205–245 g were mated with male rats (Envigo, Barcelona, Spain), and day of conception was determined by vaginal smear. Pregnant rats were divided into control dams (CON-dams; *n* = 6), fed ad libitum, and calorie-restricted dams (CR-dams; *n* = 13), exposed to a 25% calorie restriction from day 1 to 12 of gestation, with both included since epidemiological evidence in humans [36] and experimental studies in animal models [2] have suggested that the first part of gestation is especially critical in the programming of later obesity predisposition in the offspring. After delivery, litters were equated to 10 pups per dam on day 1 of lactation (5 males and 5 females, when possible). Pups from all CR-dams were divided into 4 groups: CR-L, supplemented with an oral solution of recombinant murine leptin (PeproTech, London, UK), equivalent to five times the average amount ingested normally from maternal milk, as previously described [18]; CR-M, supplemented with an oral solution of myo-inositol (Sigma-Aldrich, St. Louis, MO, USA), equivalent to two times the average amount ingested normally from maternal milk, as previously detailed [31]; CR-LM, supplemented with an oral combination of both compounds that provided animals the same amount of leptin and myo-inositol as CR-L and CR-M groups, respectively; and CR-V, treated with the vehicle (water). Pups from all CON-dams were treated with water (CON-V). The supplementation was carried out during the whole suckling period, from day 1 to 20 of lactation. The amounts of leptin and/or myo-inositol given daily to the pups are detailed in the Supplementary Material Table S2. Rats were weaned at 21 days of life, and 36 pups from CON-V group (18 males and 18 females), 32 from CR-V group (15 males and 17 females), 32 from CR-L group (15 males and 17 females), 32 from CR-M group (15 males and 17 females), and 32 from CR-LM group (15 males and 17 females) were placed 2 or 3 per cage and fed in ad libitum conditions. Body weight was measured at birth and was followed, together with food intake, from weaning to 25 days of life. Body composition was measured by EchoMRI-700 (Echo Medical Systems, LLC. Houston, TX, USA) at the age of 24 days. A few days after weaning, on day 25 of life, animals were sacrificed by decapitation during the first 2 h after the beginning of the light cycle under ad libitum feeding conditions. Day 25 (i.e., 5 days after finishing the supplementation) was chosen to study the already established programming effects of leptin and/or myo-inositol ingested during lactation, without interference of the supplementation per se. Moreover, in rats, typical circadian rhythms are already established around 25 days of life [37], allowing us to have a suitable window of time to also follow food intake of the animals. One set of animals (*n* = 9–10 per group) was used for circulating parameters determination and gene expression analysis in hypothalamus, and the other set (*n* = 5–7 per group) for PBMC isolation and morphometric and immunohistochemical studies in hypothalamic nuclei. Both plasma and PBMC samples were obtained from trunk blood. To obtain the plasma, blood was collected in heparinized tubes and subsequently centrifuged at 1000 g for 10 min, and PBMC isolation was performed as previously described [24]. For gene expression analysis, the hypothalamus was removed and stored at −80 °C until further RNA extraction, and for morphometric and immunohistochemical study, brain samples were fixed and preserved as detailed in previous studies [8,22,38].

### 2.2. Determination of Circulating Parameters

Enzyme-linked immunosorbent assay (ELISA) kits were used to determine plasma insulin and leptin levels, specifically Mercodia Rat Insulin ELISA (Mercodia AB, Uppsala, Sweden) and Rat Leptin Quantikine ELISA kit (R&D Systems, Minneapolis, MN, USA). Circulating triglycerides (TG), non-esterified fatty acids (NEFA), and myo-inositol were measured by using the following enzymatic colorimetric kits: Serum Triglyceride Determination (Sigma Diagnostics, St. Louis, MO, USA) and NEFA-HR kit (Wako Chemicals GmbH, Neuss, Germany), according to manufacturer’s instructions, and myo-Inositol Assay Kit (Megazyme Ltd., Wicklow, Ireland), as previously adapted [27]. Glucose levels were measured in fresh blood by Accu-Check Glucometer (Roche Diagnostics, Barcelona, Spain).

### 2.3. RNA Extraction

RNA was extracted from hypothalamus using TriPure Reagent (Roche Diagnostic Gmbh, Mannheim, Germany) and from PBMC using the EZNA Total RNA Kit I (Omega Bio-Tek, Inc., Nocross, GA, USA), in accordance to the manufacturer’s instructions. Quantification of total RNA was performed with the NanoDrop ND-1000 spectrophotometer (NanoDrop Technologies, Inc., Wilmington, DE, USA), and 1% agarose gel electrophoresis was carried out to confirm its integrity.

### 2.4. Real-Time Quantitative Polymerase Chain Reaction (RT-qPCR) Analysis

RT-qPCR was used to measure mRNA expression levels of leptin receptor (*Lepr*), suppressor of cytokine signalling-3 (*Socs3*), insulin receptor (*Insr*), neuropeptide Y (*Npy*), agouti-related peptide (*Agrp*), brain-derived neurotrophic factor (*Bdnf*), pro-opiomelanocortin (*Pomc*), cocaine- and amphetamine-regulated transcript (*Cart*), and melanocortin 4 receptor (*Mc4r*) in hypothalamus, and low-density lipoprotein receptor-related protein (*Lrp11*), glutaminase (*Gls*), uncoupling protein 2 (*Ucp2*), and NPC intracellular cholesterol transporter 1 (*Npc1*) in PBMC. For hypothalamus, 0.25 µg of RNA (in a final volume of 12.5 µL) were denatured at 65 °C for 10 min and reverse transcribed using MuLV reverse transcriptase (Applied Biosystems, Madrid, Spain) at 25 °C for 10 min, 37 °C for 50 min, and 70 °C for 15 min. In the case of RNA from PBMC, cDNA was obtained using the iScript cDNA Synthesis Kit (Bio-Rad Laboratories, SA, Madrid, Spain), as previously described [31]. RT-qPCR reactions were carried out from diluted cDNA template (1/10 for hypothalamus and 1/5 for PBMC), forward and reverse primers (10 μM), and Power SYBER Green PCR Master Mix (Applied Biosystems, CA, USA), using the Applied Biosystems StepOne Plus Real-time PCR Systems (Applied Biosystem). The temperature profile of the reactions was 10 min at 95 °C and 40 two-temperature cycles of 15 s at 95 °C and 1 min at 60 °C, and a melting curve was also obtained after each run to confirm the purity of the products. The threshold cycle was determined by the StepOne Software v2.2.2, and the relative expression of the analyzed genes was expressed as a percentage of CON-V male animals and using the 2^−ΔΔCt^ method. Reference genes used in this analysis were β*-actin* and guanosine diphosphate dissociation inhibitor 1 (*Gdi1*). Sequences and amplicon size of primers (Sigma, Madrid, Spain) are detailed in Supplementary Material Table S1.

### 2.5. Morphometric and Immunohistochemical Analysis

Blocks containing the hypothalamus were obtained as previously described [22], and 5-µm sections were progressively cut with a microtome and mounted in Super Frost/Plus slides until reaching the paraventricular nucleus (PVN) zone first and the arcuate nucleus (ARC) later, according to previously published coordinates [22,39]. Hematoxylin/eosin staining was used to verify that areas of interest were reached, and Nissl staining was subsequently performed as described [39] for the specific count of neurons in PVN and ARC. Immunohistochemical demonstration of neuropeptide Y positive (NPY^+^) and α-melanocyte-stimulating hormones positive (αMSH^+^) neurons in the ARC was carried out as previously described [22], using polyclonal rabbit anti-NPY antibody (Sigma-Aldrich, St. Louis, MO, USA, Catalog number N9528, 1:1000 in PBS 0.1% Triton X-100 with 1% BSA for 1 h and 15 min at 37 °C) and polyclonal rabbit anti-MSH alpha antibody (Biorbyt Ltd., Cambridge, UK, Catalog number orb13589, 1:100 in PBS 0.1% Triton X-100 with 1% BSA overnight at 4 °C). Negative controls were carried out by omission of primary antibody. Images from light microscopy were digitalized. The ARC was drawn first in each Nissl-stained section, and the number of stained neurons was subsequently measured using AxioVision40V 4.6.3.0. software (Carl Zeiss, Imaging Solutions GmbH, Munich, Germany). In addition, the number of NPY^+^ and αMSH^+^ neurons were also counted in the image obtained from the corresponding slide and taking the Nissl-stained section as a reference. The described images analysis from all groups was performed in a blind fashion.

### 2.6. Statistical Analysis

Data are represented as the mean ± s.e.m. Differences between sexes and groups (CON-V, CR-V, CR-L, CR-M, and CR-LM) were assessed by two-way ANOVA, followed by least significance difference (LSD) post-hoc test in the case of a significant group effect. With interactive effect, differences between groups within each sex were analyzed by one-way ANOVA, followed by LSD post-hoc test. Single comparisons between groups were assessed by Mann–Whitney U test. Normality and homogeneity of variances of the data were performed by the Shapiro–Wilk and the Bartlett test, respectively. Relation between two variables was assessed by the Pearson’s correlation coefficient. When necessary, logarithmic transformation of the data was carried out. Analyses were performed with SPSS for Windows (SPSS, Chicago, IL, USA), defining the threshold of significance at *p* < 0.05.

## 3. Results

### 3.1. Food Intake and Body-Weight Related Parameters

Data of food intake and body weight and body composition are summarized in Table 1. Male and female CR rats displayed lower body weight than their respective CON-V groups at both weaning and sacrifice (postnatal days 21 and 25) (two-way ANOVA), but no differences were found at birth. Body weight gain from weaning to sacrifice was significantly lower in CR-V rats with respect to CON-V rats (two-way ANOVA). This parameter was normalized to that of control in animals treated with leptin or myo-inositol but not in those treated with the combination. No significant differences between groups were found regarding growth rate from weaning to sacrifice (expressed relative to the body weight of the animals at weaning) (data not shown). Body weight at sacrifice and body weight gain and growth rate from weaning to sacrifice were higher in males than in females (two-way ANOVA). Cumulative food intake from postnatal day 21 to 25 was lower in CR rats compared to controls and partially normalized in animals supplemented with myo-inositol but not in those treated with leptin or the combination (one-way ANOVA). No differences were found between groups regarding body composition, but females showed higher fat mass percentage than males (two-way ANOVA).

### 3.2. Circulating Parameters

Circulating levels of TG, NEFA, myo-inositol, glucose, insulin, and leptin in 25-day-old male and female rats are shown in Table 2. NEFA and myo-inositol levels were significantly lower in all male and female CR groups compared to CON-V animals (two-way ANOVA). CR-M animals showed higher insulin plasma levels than CON-V and CR-V groups (two-way ANOVA). All CR male groups but not females displayed lower plasma leptin levels compared to CON-V males (interactive effect between sex and group, two-way ANOVA; and one-way ANOVA). In addition, CR-LM females displayed significantly lower circulating leptin levels than CR-V animals (Mann–Whitney U test). No significant differences were found regarding TG and glucose levels.

### 3.3. Morphometric and Immunohistochemical Analysis of Hypothalamic Nuclei

**Figure 1:**
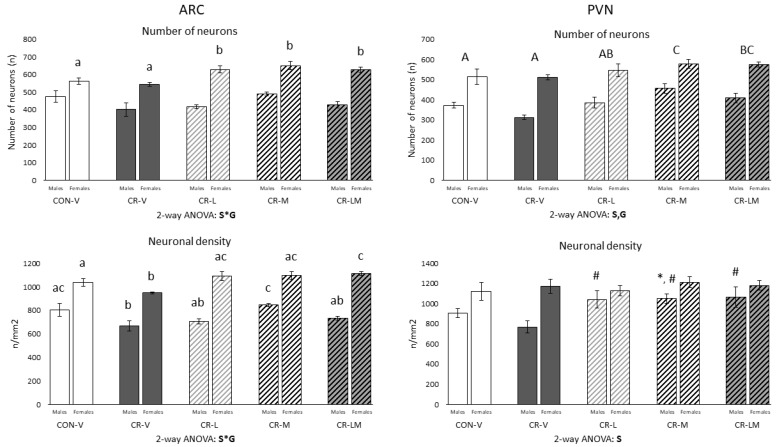
Number of neurons and neuronal density in the arcuate (ARC) and paraventricular (PVN) nuclei of the hypothalamus of 25-day-old male and female offspring of control (CON) and 25% gestational calorie-restricted (CR) dams. Data are means ± s.e.m (*n* = 5–7). Statistics: Two-way ANOVA was performed to assess the effects of sex and group (CON-V, CR-V, CR-L, CR-M, and CR-LM). One-way ANOVA was carried out to determine differences between groups in each sex separately when previous two-way ANOVA showed interactive effect between sex and group. ANOVA was followed by a least significant difference (LSD) post-hoc test. Mann–Whitney U test was used for single comparisons between groups. Symbols: S, effect of sex; G, effect of group; S*G, interactive effect between sex and group; A ≠ B ≠ C (*p* < 0.05, two-way ANOVA); a ≠ b ≠ c (*p* < 0.05, one-way ANOVA); *, different from their respective CON-V group; #, different from their respective CR-V group (*p* < 0.05, Mann–Whitney U test). Abbreviations: vehicle (V), leptin (L), myo-inositol (M), leptin and myo-inositol (LM).

Morphometric studies were performed in the ARC and PVN of 25-day-old rats (Figure 1). Notably, leptin and myo-inositol treatments, alone or in combination, resulted in changes in the total number of neurons in the ARC and PVN nuclei with respect to those treated with the vehicle, both CON-V and CR-V groups. In the ARC, females but not males treated with leptin, myo-inositol, or the combination showed a higher number of total neurons in comparison to CON-V and CR-V males (interactive effect between sex and group, two-way ANOVA; and one-way ANOVA). In the PVN, both male and female rats treated with myo-inositol or the combination displayed a greater number of total neurons than CON-V and CR-V rats (two-way ANOVA). Neuronal density was also affected by the treatments during lactation in both nuclei. Gestational CR caused a diminution of the neuronal density in the ARC compared to controls in both male and female rats, which was reverted by the treatments in a sex-dependent manner (interactive effect between sex and group, two-way ANOVA; and one-way ANOVA). In females, leptin and myo-inositol fully restored the neuronal density to the values of the control group, while the combination increased it even beyond the control group levels (one-way ANOVA). In males, myo-inositol treatment totally restored neuronal density to control values, whereas the reversion was partial in the groups supplemented with leptin or the combination (one-way ANOVA). In the PVN, males supplemented with leptin, myo-inositol, or the combination displayed higher neuronal density than CR-V animals, and those treated with myo-inositol also displayed a higher value than CON-V animals (Mann–Whitney U test), while no significant differences were observed between CR-V and CON-V groups. Of note, female rats presented a higher number of neurons and a higher neuronal density in the PVN than males (two-way ANOVA).

**Figure 2:**
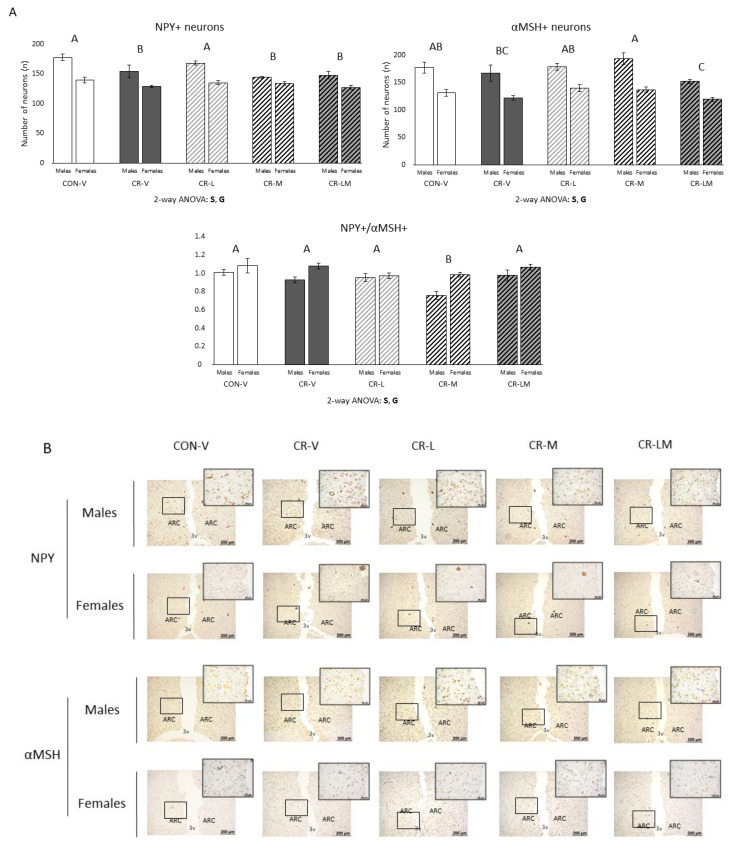
(**A**). Number of NPY and αMSH positive (NPY^+^ and αMSH^+^) neurons, together with NPY^+^/αMSH^+^ ratio, in the arcuate nucleus (ARC) of the hypothalamus of 25-day-old male and female offspring of control (CON) and 25% gestational calorie-restricted (CR) dams. Data are means ± s.e.m (*n* = 4–6). Statistics: Two-way ANOVA was performed to assess the effects of sex and group (CON-V, CR-V, CR-L, CR-M, and CR-LM), followed by a least significant difference (LSD) post-hoc test. Symbols: S, effect of sex; G, effect of group; A ≠ B ≠ C (*p* < 0.05, two-way ANOVA). (**B**). Representative brain sections immunostained for NPY and αMSH. Insets: enlargement of the corresponding framed areas. Abbreviations: vehicle (V), leptin (L), myo-inositol (M), leptin and myo-inositol (LM), third ventricle (3v). Scale bar: 200 µm and 50 µm for insets.

Immunohistochemical analyses of NPY^+^ and αMSH^+^ neurons were carried out in the ARC of 25-day-old rats (Figure 2). Male and female CR-V rats showed a reduction in the number of NPY^+^ neurons, which was restored by leptin supplementation but not by myo-inositol or the combination (two-way ANOVA). Regarding αMSH^+^ neurons, myo-inositol supplementation led to a higher number of αMSH^+^ neurons compared to the CR-V group, whereas the combination resulted in lower number of αMSH^+^ neurons than control animals (two-way ANOVA). The ratio of NPY^+^ to αMSH^+^ neurons was calculated as an indicator of the satiety neuronal balance. Interestingly, male and female CR-M rats presented a lower NPY^+^/αMSH^+^ ratio in comparison to the other groups of animals (two-way ANOVA). Female rats showed less NPY^+^ and αMSH^+^ neurons but higher NPY^+^/αMSH^+^ ratio than males (two-way ANOVA). Percentage of NPY^+^ and αMSH^+^ neurons with respect to the total number of neurons was also calculated (Supplementary Material Figure S1). No effect of gestational calorie restriction was observed in any case, but myo-inositol supplementation in males and leptin and/or myo-inositol supplementation in females resulted in a decreased percentage of NPY^+^ neurons with respect to the CON-V, CR-V, and CR-L groups (in the case of males) and with respect to the CON-V and CR-V groups (in the case of females) (one-way ANOVA). Myo-inositol treatment, alone and in combination with leptin, also led to a reduction in the percentage of αMSH^+^ neurons with respect to the CR-V and CR-L groups, especially evident in males (two-way ANOVA).

### 3.4. mRNA Expression Levels in Hypothalamus

**Figure 3:**
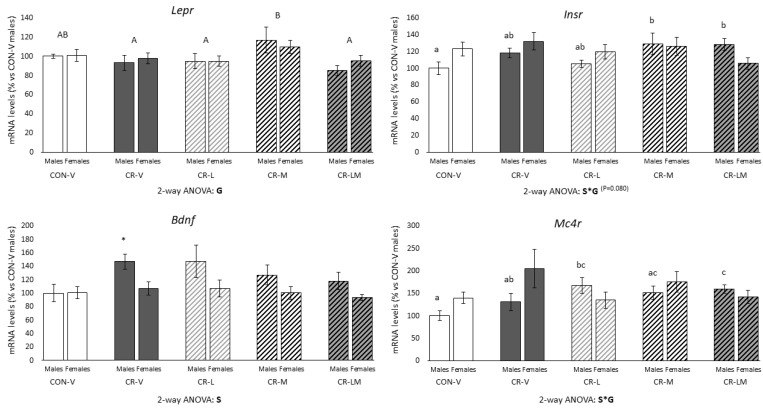
Hypothalamic expression levels of selected genes in 25-day-old male and female offspring of control (CON) and 25% gestational calorie-restricted (CR) dams. mRNA levels are expressed as a percentage of the value of CON-V male rats. Data are means ± s.e.m (*n* = 8–10). Statistics: Two-way ANOVA was performed to assess the effects of sex and group (CON-V, CR-V, CR-L, CR-M, and CR-LM). One-way ANOVA was carried out to determine differences between groups in each sex separately when previous two-way ANOVA showed interactive effect between sex and group. ANOVA was followed by a least significant difference (LSD) post-hoc test. Mann–Whitney U test was used for single comparisons between groups. Symbols: S, effect of sex; G, effect of group; S*G, interactive effect between sex and group; A ≠ B (*p* < 0.05, two-way ANOVA); a ≠ b ≠ c (*p* < 0.05, one-way ANOVA); *, different from their respective CON-V group (*p* < 0.05, Mann–Whitney U test). Abbreviations: vehicle (V), leptin (L), myo-inositol (M), leptin and myo-inositol (LM).

Figure 3 shows mRNA expression levels of selected genes in the hypothalamus of 25-day-old male and female rats. These genes were chosen for their role in energy homeostasis and/or because previous studies have shown both alterations in their expression due to gestational calorie restriction and/or a response to leptin treatment during lactation [8,22,40]. Interestingly, both male and female rats treated with myo-inositol during the suckling period showed higher *Lepr* mRNA levels in comparison with CR-V, CR-L, and CR-LM animals (two-way ANOVA). CR-V male animals but not those supplemented with leptin, myo-inositol, or the combination showed increased *Bdnf* mRNA levels compared to CON-V rats (Mann–Whitney U test). A gender-dependent effect was observed regarding *Insr* and *Mc4r* mRNA expression levels due to the gestational conditions or the treatment (interactive effect between sex and group, with *p* = 0.080 for Insr, two-way ANOVA; and one-way ANOVA). CR males supplemented with myo-inositol or the combination showed higher mRNA levels of *Insr* with respect to the control group (one-way ANOVA). In turn, CR males supplemented with leptin or the combination displayed higher *Mc4r* mRNA levels in comparison to the control group (one-way ANOVA). CR-LM males also showed higher *Mc4r* mRNA levels than the CR-V group (one-way ANOVA). No significant differences were found in females regarding expression levels of *Insr* and *Mc4r*. No significant differences were found in the expression levels of *Socs3*, *Npy*, *Agrp*, *Pomc,* and *Cart* due gestational conditions or the treatments during the suckling period (data not shown). Expression levels of *Bdnf* and of *Agrp* (data not shown) were significantly lower in females than in males (two-way ANOVA). Notably, in males, expression levels of *Insr* were correlated with circulating insulin and myo-inositol levels (r = 0.407, *p* = 0.010 and r = −0.476, *p* = 0.001, respectively), whereas in females, expression levels of *Cart* and *Mc4r* were correlated with circulating leptin levels (r = 0.286, *p* = 0.046 and r = 0.317, *p* = 0.027, respectively).

### 3.5. mRNA Expression Levels in PBMC

**Figure 4:**
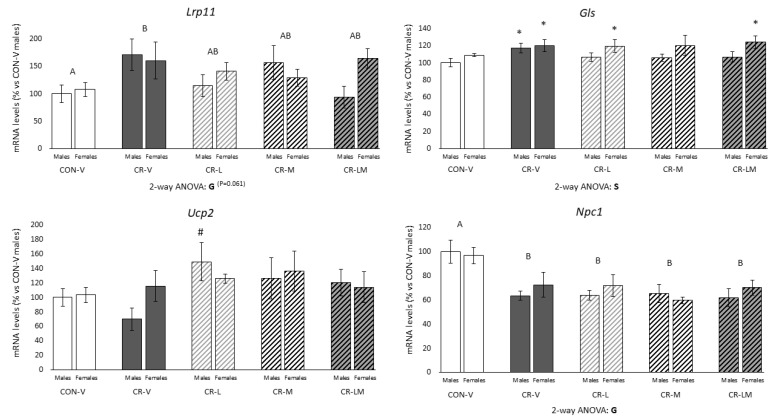
Expression levels of selected genes in peripheral blood mononuclear cells (PBMC) of 25-day-old male and female offspring of control (CON) and 25% gestational calorie-restricted (CR) dams. mRNA levels are expressed as a percentage of the value of CON-V male rats. Data are means ± s.e.m (*n* = 6–9). Statistics: Two-way ANOVA was performed to assess the effects of sex and group (CON-V, CR-V, CR-L, CR-M, and CR-LM), followed by a least significant difference (LSD) post-hoc test. Mann–Whitney U test was used for single comparisons between groups. Symbols: S, effect of sex; G, effect of group; A ≠ B (*p* < 0.05, two-way ANOVA); *, different from their respective CON-V group; #, different from their respective CR-V group (*p* < 0.05, Mann–Whitney U test). Abbreviations: vehicle (V), leptin (L), myo-inositol (M), leptin and myo-inositol (LM).

mRNA expression levels of selected genes in PBMC of 25-day-old male and female rats are shown in Figure 4. mRNA levels of *Lrp11* and *Gls* were increased in CR-V male and female rats and partially normalized to CON-V levels in CR-L, CR-M, and CR-LM animals in both sexes for *Lrp11* (*p* = 0.061, two-way ANOVA) but only in males for *Gls* (Mann–Whitney U test). CR-V, CR-L, and CR-LM females displayed higher *Gls* mRNA levels than CON-V females (Mann–Whitney U test). Notably, CR male rats supplemented with leptin showed higher *Ucp2* mRNA levels than CR males treated with the vehicle (Mann–Whitney U test). *Npc1* mRNA levels were reduced in all male and female CR groups regardless of leptin and/or myo-inositol supplementation during the suckling period (two-way ANOVA). No significant differences were found in the expression levels of *Ubash3b*, *Gla*, *Paox,* and *Tmsb4x* (data not shown).

## 4. Discussion

Lactation represents a critical window of development, and adequate/optimal nutrition during this period may be overriding for the future metabolic health of the offspring, including the prevention of possible harmful programming effects due to adverse fetal conditions [41]. There is compelling evidence from intervention studies in animal models on the essential role of leptin ingested during lactation in the prevention of obesity and other related pathologies in adulthood since it ensures a correct programming and development of hypothalamic structures involved in energy metabolism [16]. Recent studies also point to myo-inositol as a milk component of potential interest since its supplementation at physiological doses during the suckling period has shown beneficial effects on the prevention of insulin resistance and hypertriglyceridemia in male offspring of calorie-restricted rats when exposed to an obesogenic diet in adulthood [31]. However, short-term programming effects of myo-inositol during lactation and whether it may also affect hypothalamic circuitry involved in energy homeostasis are still unknown. Therefore, here, we have evaluated the effects of myo-inositol supplementation, alone and in combination with leptin, during the suckling period in 25-day-old male and female rats exposed to mild/moderate gestational calorie restriction on hypothalamic structures and the expression profile of early potential biomarkers of metabolic health in PBMC.

Mild/moderate gestational calorie restriction has been widely related to detrimental effects on later body weight and adiposity of the offspring [2]. Nevertheless, studies in rodent models show variability in body weight-related outputs of this metabolic programming model [42], probably due a myriad of factors, including the sex and the severity, type, and timing of the restriction [2,43]. Here, all groups of pups that underwent gestational undernutrition displayed lower body weight at both weaning and sacrifice compared to control animals, according to previous studies [22]. We did not measure energy intake during lactation, but lower energy intake after weaning in all CR groups in comparison to controls was observed with the exception of animals supplemented with myo-inositol, which showed a partial normalization to control values. Of note, myo-inositol-supplemented animals also displayed a higher body weight gain than CR-V rats from weaning (postnatal day 21) to sacrifice (postnatal day 25).

Nutrient deprivation during gestation also affected circulating parameters at the age of 25 days, probably reflecting lower body weight. Specifically, CR rats displayed reduced NEFA and myo-inositol levels in males and females and lower leptin levels in male rats compared to the CON-V group and regardless of leptin and/or myo-inositol supplementation during lactation. Reduced leptin levels have also been reported in the male progeny of 20% gestational calorie-restricted rats [8,22,44].

Hypothalamic circuitry is largely established during early development [45]. Thus, this period is particularly sensitive to the effects of maternal undernutrition (20–25% energy intake reduction) during pregnancy, leading to perturbations in the appetite and energy expenditure regulatory systems of the offspring by affecting the development of hypothalamic structures [45]. We have previously observed important sex differences [8,22]. Likewise, here, the offspring of 25% calorie-restricted dams during pregnancy and treated with the vehicle, both males and females, also displayed a significant reduction in the neuronal density of the ARC compared to controls. The supplementation with physiological doses of both leptin and myo-inositol, individually or in combination, during lactation led to a higher number of neurons in the ARC (females) in comparison with those treated with the vehicle (both CON-V and CR-V groups) and restored its neuronal density (both sexes). Regarding the PVN, male and female rats treated with myo-inositol, alone or combined with leptin, displayed a higher number of neurons than those pups treated with the vehicle (control and CR groups). Additionally, in this line, male rats treated with leptin, myo-inositol, or the combination during lactation displayed higher neuronal density than CR-V animals. Taken together, these results could be reflecting a programming effect of myo-inositol during the suckling period on the development of hypothalamic nuclei involved in food intake and energy expenditure and comparable to the effects of leptin. No additive effect of both compounds has been observed.

The effects of leptin and myo-inositol supplementation in the ARC were further studied by immunohistochemical analysis of orexigenic and anorexigenic neurons. Of note, female rats exhibited less number and percentage of NPY^+^ and αMSH^+^ neurons than males regardless the type of treatment. Sex differences in the abundance of neurons in the hypothalamus, particularly the presence of less NPY-expressing cells and lower NPY levels in females compared with males, have been previously described in rodents [46,47]. According to our previous results [8], male and female CR-V rats showed a reduction in the number of NPY^+^ neurons in comparison to controls. Notably, leptin treatment was able to normalize the number of NPY^+^ cells to control levels, as previously described [22], but no effects were observed with myo-inositol treatment or the combination. In fact, the percentage of NPY^+^ neurons decreased in males by myo-inositol treatment with respect to the CR-V group and by all treatments in females, which can be attributed to the increase in the total number of neurons. However, which type of neurons are mainly affected other than those determined here has not been studied. Regarding αMSH^+^ neurons, although we observed no significant effects of gestational calorie restriction, also according to previous results [8], supplementation with myo-inositol but not with leptin or the combination resulted in a significant increase in the number of αMSH^+^ neurons in both male and female animals in comparison with the CR-V group. However, this was not translated into a greater percentage of αMSH^+^ neurons in comparison with the CR-V group but the opposite due to the greater effect of myo-inositol increasing the total number of neurons in the ARC, particularly in females. Of note, CR-M animals displayed a significant decrease of the NPY^+^/αMSH^+^ ratio. These results suggest a better control of food intake and energy balance at an early age, which may be tentatively related with the relative normalization of energy intake after weaning in animals treated with myo-inositol to values similar to controls.

Early programming of leptin resistance and insulin resistance is considered one of the main causes of metabolic dysregulation in later stages of life [48,49]. Impaired leptin and insulin sensitivity has been described in animals exposed to inadequate nutritional conditions during gestation [44]. Notably, here, we show that both male and female rats exposed to gestational calorie restriction displayed higher expression levels of *Lepr* when supplemented with physiological doses of myo-inositol during lactation. This could suggest that these animals, despite being exposed to an adverse nutritional condition during fetal life, are more sensitive to the central action of leptin and, therefore, may be more protected against the development of obesity and its related comorbidities in adulthood. An increased central leptin sensitivity has been effectively described in adult male rats treated with physiological doses of leptin during the suckling period [18,21]. Therefore, the increased sensitivity to leptin action during early age in myo-inositol supplemented animals may tentatively explain, at least in part, the similar effects observed with both treatments, leptin and myo-inositol, when administered individually, on neuronal development. It could be hypothesized that the possible neurotrophic action observed with myo-inositol supplementation during lactation could be explained, in part, by a myo-inositol-mediated empowered action of leptin (concomitant to increased sensitivity to this hormone) naturally ingested with milk. However, this hypothesis needs to be further explored, and a direct effect of myo-inositol cannot be ruled out.

Besides the effects on leptin receptor, myo-inositol treatment both alone and combined with leptin also increased hypothalamic *Insr* mRNA expression of calorie-restricted male pups. In this line, it is noteworthy that myo-inositol supplemented rats also displayed significantly higher plasma insulin levels. Therefore, the presence of increased circulating insulin levels together with increased hypothalamic expression of the insulin receptor gene could be reflecting an increased insulin action in CR animals treated with myo-inositol. In fact, a positive correlation between circulating levels of insulin and *Insr* mRNA levels has been found, particularly in male rats. These data on the early effects of myo-inositol on insulin signaling are in accordance with our previous finding showing that male animals treated with myo-inositol during lactation are more protected against the development of insulin resistance and hypertriglyceridemia after a diabetogenic diet in adulthood [31]. Contrarily, circulating myo-inositol levels were negatively correlated with hypothalamic *Insr* expression in males. This might seem contradictory, but it must be considered that circulating myo-inositol (measured five days after the end of the supplementation period) was decreased in all groups exposed to gestational calorie restriction regardless the type of supplementation. These results might indicate that the potential positive effects of myo-inositol supplementation on hypothalamic *Insr* expression might not be directly dependent on the circulating levels at this time period after weaning. Therefore, the study of possible mediators for the action of myo-inositol ingested by the suckling pups, together with the sex differences, might be of interest. These sex-specific results (only in males) might also be associated with the suggested more sensitivity to insulin in male brain than in females [50].

Despite changes described in leptin and insulin receptor, expression levels of other genes related to the action of these hormones showed no significant differences between groups in the hypothalamus with the exception of *Mc4r* and *Bdnf*. Hypothalamic BDNF is an anorectic factor and a regulator of energy expenditure apart from a main neurotrophin, therefore regulating neuronal development [40,51]. The only significant change observed here was that CR males treated with the vehicle displayed a significant increase of *Bdnf* mRNA levels compared to control animals, which might reflect a compensatory response. Of note, in females, despite that there were no differences in *Bdnf* expression between groups, their expression levels were negatively correlated with the growth rate of animals from weaning to sacrifice (r = −0.295, *p* = 0.044), which might be in accordance with its anorexigenic action. MC4R has a key role coordinating energy balance, glucose homeostasis, and sympathetic nerve activity [52], and impaired MC4R function has been related with overfeeding and the development obesity and associated metabolic alterations in animal models and humans [53,54]. Thus, the present results on increased *Mc4r* mRNA levels in males exposed to gestational calorie restriction and supplemented with leptin or the combined treatment could explain, at least in part, the lower energy intake of these animals compared to the control ones and could be acting as a protective mechanism to counteract the programmed predisposition to insulin resistance due to fetal adverse conditions. In addition, the positive correlation between circulating leptin and hypothalamic expression levels of *Mc4r* as well as of *Cart* only found in females could be in agreement with the reported higher female sensitivity to the action of leptin in the brain, whereas males appear to be more sensitive to insulin (as stated above) [49].

Besides the study of hypothalamus as a key tissue in energy homeostasis, we considered of interest to study the expression levels of selected genes, previously identified as candidate early markers of metabolic health in PBMC, as a minimally invasive surrogate tissue [24]. In this sense, increased mRNA levels of *Lrp11* and *Gls* in PBMC were previously proposed as candidate early biomarkers of impaired metabolic health associated to the effects of mild/moderate gestational calorie restriction in rats, and they were sensitive to adequate reprograming by early life intervention [24]. Here, expression levels of *Lrp11* and *Gls* were also increased in male and female offspring of rats exposed to gestational undernutrition and treated with the vehicle in comparison to their respective controls, supporting our previous results [24]. Interestingly leptin and myo-inositol supplementation both alone or in combination partially normalized the expression levels of *Lrp11* in males and females. *Gls* mRNA levels were also normalized in CR males treated with leptin, myo-inositol, or the combination and in females treated with myo-inositol. Thus, our results reinforce the potential of both compounds, leptin and myo-inositol, administered separately during the suckling period, to normalize, at least in part, the programming effects associated with adverse conditions during pregnancy, while also supporting the utility of transcript levels of *Lrp11* and *Gls* in PBMC as early biomarkers of impaired metabolic health due to unfavorable conditions during gestation and its reversion by adequate/optimal nutrition during lactation.

Expression levels of *Ucp2* in peripheral blood cells (PBC) have also been proposed as an early potential biomarker of metabolic status in children [34]. Specifically, a downregulation of this gene was described in formula-fed infants compared to breast-fed ones. Here, leptin supplementation during the suckling period resulted in a significant increment of *Ucp2* mRNA levels in males but not in females compared to CR males treated with the vehicle, a fact that would be in agreement with the described beneficial effects of postnatal leptin supplementation in male rats [18]. The gender-dependent effect in *Ucp2* expression levels has also been described in humans; Priego et al. described reduced *Ucp2* expression levels in the PBC of formula-fed male infants in comparison with breast-fed ones but not in females [55]. In addition, upregulation of *Ucp2* mRNA levels in breast-fed vs. formula-fed infants was more marked in those born small for gestational age compared to children with adequate birth weight [34]. Therefore, it could be speculated that *Ucp2* expression in PBMC may be reflecting adequate reprogramming of metabolic disturbances caused by adverse intrauterine condition by an optimal nutrition during the suckling period, especially in males.

mRNA levels of *Npc1* in blood cells have been previously proposed as a potential early biomarker of increased risk of developing insulin resistance [35]. Particularly, reduced *Npc1* expression levels have been described in blood cells of adult humans with mildly impaired metabolic health and in adult rats exposed to mild/moderate calorie restriction during gestation [35]. In this sense, different studies have concluded on the association between NPC1 protein deficiency and metabolic features associated with the impairment of insulin signaling pathways, such as hyperleptinemia and dyslipidemia [56,57]. In the present study, performed at an early age, *Npc1* expression levels were significantly lower in all groups of male and female descendants exposed to mild/moderate calorie restriction during gestation regardless of the treatment that these animals received during the suckling period. Considering the involvement of this gene in lipid and glucose metabolism pathways and therefore in the development of obesity and its related alterations, it could be highlighted the interest of the *Npc1* mRNA levels in blood cells as a potential early biomarker of impaired conditions during gestation regardless of the possible reversal by conditions during lactation. In fact, in our previous study, *Npc1* expression levels in PBMC of adult rats were found to be normalized in CR male rats treated with leptin during the suckling period, in accordance with phenotype normalization [35].

In conclusion, supplementation with physiological doses of myo-inositol during lactation in rats, alone and in combination with leptin, leads to partially comparable effects to those shown for leptin supplementation during lactation on the reversion of alterations programmed by an inadequate fetal environment on the hypothalamic structure and function in a sex-dependent manner. Particularly, myo-inositol supplementation in the offspring of gestational calorie-restricted dams leads to an increased number of neurons in the ARC (especially in females) and PVN nuclei and to increased αMSH^+^ neurons in the ARC along with increased hypothalamic expression levels of *Insr* (in males) and *Lepr*. To the best of our knowledge, this is the first time that myo-inositol supplementation during lactation is shown to have effects on hypothalamic development in rats, so it would be also interesting to study its potential effects on the offspring of well-nourished dams during gestation. Whether this is a direct effect or the result of an empowered leptin action needs to be further studied. Of note, the present results do not generally show an additive, subtractive, or beneficial effect of the combined supplementation with leptin and myo-inositol during lactation on the offspring health, although possible effects in the longer term and/or in other parameters that have not been analyzed here cannot be ruled out. In addition, our findings reinforce the usefulness of expression levels of selected genes in PBMC, specifically *Lrp11*, *Gls*, and *Ucp*, as early biomarkers of impaired metabolic health due to gestational calorie restriction, and its potential reversion by adequate treatment during lactation, as shown with leptin, myo-inositol, or the combination.

## Figures and Tables

**Table 1:** Food intake and body weight and composition related measurements of 25-day-old male and female offspring of control (CON) and 25% gestational calorie-restricted (CR) dams. Data are means ± s.e.m (*n* = 15–18). Statistics: Two-way ANOVA was performed to assess the effects of sex and group (CON-V, CR-V, CR-L, CR-M, and CR-LM). One-way ANOVA was carried out to determine differences between groups. ANOVA was followed by a least significant difference (LSD) post-hoc test. Symbols: S, effect of sex; G, effect of group; A ≠ B ≠ C (*p* < 0.05, two-way ANOVA); a ≠ b (*p* < 0.05, one-way ANOVA). Abbreviations: vehicle (V), leptin (L), myo-inositol (M), leptin and myo-inositol (LM).

	CON-V	CR-V	CR-L	CR-M	CR-LM	2-Way ANOVA
**Body weight (g)**						
At birth (d1)						
Males	7.1 ± 0.1	6.9 ± 0.3	6.8 ± 0.1	6.9 ± 0.1	6.8 ± 0.2	
Females	6.7 ± 0.1	6.7 ± 0.2	6.7 ± 0.2	6.8 ± 0.2	6.8 ± 0.2	
At weaning (d21)	A	B	B	B	B	
Males	51.1 ± 1.0	48.0 ± 1.0	47.9 ± 0.9	48.0 ± 1.2	47.9 ± 1.1	G
Females	49.6 ± 1.1	46.5 ± 0.9	47.5 ± 0.8	47.6 ± 0.5	47.6 ± 1.0	
At sacrifice (d25)	A	B	B	B	B	
Males	68.5 ± 1.6	63.7 ± 1.4	64.7 ± 1.2	65.5 ± 1.3	63.4 ± 1.7	S, G
Females	65.6 ± 1.3	61.4 ± 1.2	62.2 ± 1.1	63.8 ± 0.7	62.9 ± 1.3	
**Body weight gain (from d21 to d25, g)**	A	B	ABC	AC	BC	
Males	17.5 ± 0.9	15.6 ± 1.2	16.8 ± 0.5	17.2 ± 0.6	15.6 ± 0.7	S, G
Females	16.0 ± 0.4	14.9 ± 0.7	14.8 ± 0.4	16.2 ± 0.4	15.2 ± 0.4	
**Body composition (d24)**						
Fat mass (%)						
Males	8.9 ± 0.4	8.1 ± 0.3	8.5 ± 0.3	8.5 ± 0.2	8.1 ± 0.3	S
Females	8.6 ± 0.3	9.1 ± 0.3	9.2 ± 0.3	9.2 ± 0.3	8.7 ± 0.3	
Lean mass (%)						
Males	92.5 ± 0.7	92.1 ± 0.6	92.3 ± 0.3	93.2 ± 0.8	92.7 ± 0.3	-
Females	92.5 ± 0.6	92.3 ± 0.6	91.7 ± 0.4	91.6 ± 0.3	92.4 ± 0.4	
**Cumulative food intake (kcal)**	115 ± 3a	103 ± 2b	107 ± 3b	109 ± 2ab	104 ± 3b	

**Table 2:** Circulating triglyceride, NEFA, myo-inositol, glucose, insulin, and leptin levels of 25-day-old male and female offspring of control (CON) and 25% gestational calorie-restricted (CR) dams. Data are means ± s.e.m (*n* = 9–10). Statistics: Two-way ANOVA was performed to assess the effects of sex and group (CON-V, CR-V, CR-L, CR-M, and CR-LM). One-way ANOVA was carried out to determine differences between groups in each sex separately when previous two-way ANOVA showed interactive effect between sex and group. ANOVA was followed by a least significant difference (LSD) post-hoc test. Mann–Whitney U test was used for single comparisons between groups. Symbols: G, effect of group; S*G, interactive effect between sex and group; A ≠ B (*p* < 0.05, two-way ANOVA); a ≠ b (*p* < 0.05, one-way ANOVA); #, different from their respective CR-V group (*p* < 0.05, Mann–Whitney U test). Abbreviations: vehicle (V), leptin (L), myo-inositol (M), leptin and myo-inositol (LM).

	CON-V	CR-V	CR-L	CR-M	CR-LM	2-Way ANOVA
**Triglycerides (mg·mL^−1^)**						
Males	0.895 ± 0.055	0.928 ± 0.082	0.882 ± 0.119	0.961 ± 0.071	0.907 ± 0.084	-
Females	1.108 ± 0.131	0.909 ± 0.086	0.919 ± 0.096	0.872 ± 0.101	0.849 ± 0.090	
**NEFA (mmol·L^−1^)**	A	B	B	B	B	
Males	0.317 ± 0.048	0.241 ± 0.023	0.283 ± 0.031	0.232 ± 0.013	0.244 ± 0.026	G
Females	0.385 ± 0.053	0.231 ± 0.016	0.227 ± 0.034	0.239 ± 0.015	0.276 ± 0.036	
**Myo-inositol (µg·mL^−1^)**	A	B	B	B	B	
Males	97.2 ± 14.0	80.4 ± 7.9	70.5 ± 6.3	69.7 ± 5.3	72.1 ± 9.6	G
Females	127.1 ± 20.2	67.1 ± 3.6	65.8 ± 7.6	69.7 ± 8.2	72.3 ± 9.2	
**Glucose (mg·dL^−1^)**						
Males	129 ± 2	128 ± 3	134 ± 2	127 ± 2	127 ± 4	
Females	127 ± 2	125 ± 2	128 ± 2	127 ± 2	129 ± 2	
**Insulin (µg·L^−1^)**	A	A	AB	B	A	
Males	0.270 ± 0.050	0.185 ± 0.029	0.201 ± 0.025	0.375 ± 0.084	0.264 ± 0.041	G
Females	0.223 ± 0.049	0.334 ± 0.070	0.369 ± 0.067	0.458 ± 0.126	0.191 ± 0.020	
**Leptin (µg·L^−1^)**						
Males	2.02 ± 0.13a	1.32 ± 0.11b	1.24 ± 0.16b	1.62 ± 0.15b	1.35 ± 0.16b	S*G
Females	1.34 ± 0.22	1.48 ± 0.08	1.26 ± 0.11	1.41 ± 0.08	1.11 ± 0.06 #	

## Data Availability

The data that support the findings of this study are available from the corresponding author upon reasonable request.

## Supplementary Materials

The following are available online at https://www.mdpi.com/article/10.3390/nu13093257/s1, Table S1. Nucleotide sequences of primers used for PCR amplification. Table S2. Daily amounts of supplemented leptin and myo-inositol from day 1 to day 20 of lactation. Figure S1. Percentage of NPY and αMSH positive neurons in the arcuate nucleus of the hypothalamus.
