# Supplementary material for: Myo-Inositol Supplementation in Suckling Rats Protects against Adverse Programming Outcomes on Hypothalamic Structure Caused by Mild Gestational Calorie Restriction, Partially Comparable to Leptin Effects
Source: Nutrients. 2021 Sep 18;13(9):3257. doi: 10.3390/nu13093257 (PMC8466200; doi:10.3390/nu13093257)
Supplement: Supplementary file 1 [file nutrients-13-03257-s001.zip › nutrients-1336511-supplementary.pdf]

Supplementary Materials

Table S1. Nucleotide sequences of primers used for PCR amplification

Gene	Forward primer (5' to 3')	Reverse primer (5' to 3')	Amplicon size (pb)
β-*actin*	GAAGCTGTGCTATGTTGCCC	GGATTCCATACCCAGGAAGG	184
Gdi1	CCGCACAAGGCAAATACATC	GACTCTCTGAACCGTCATCAA	210
Lepr	AGCCAAACAAAAGCACCATT	TCCTGAGCCATCCAGTCTCT	174
Socs3	ACTGAGCCGACCTCTCTCCT	CCCCTCTGACCCTTTCTTTG	172
Insr	GTCCGGCGTTCATCAGAG	CTCCTGGGATTCATGCTGTT	242
Npy	TGGACTGACCCTCGCTCTAT	GTGTCTCAGGGCTGGATCTC	188
Agrp	AGAGTTCTCAGGTCTAAGTCT	CTTGAAGAAGCGGCAGTAGCACGT	210
Bdnf	ATTAGCGAGTGGGTCACAGC	CGAGTTCCAGTGCCTTTTGT	189
Pomc	CCTGTGAAGGTGTACCCCAATGTC	CACGTTCTTGATGATGGCGTTC	266
Cart	AGAAGAAGTACGGCCAAGTCC	CACACAGCTTCCCGATCC	84
Mc4r	TATGGTACTGGAGCGCGTAA	TCAGACGGAGGATGCTATGA	370
Lrp11	ACAGACGACCACGCCATT	CCTGGGAAGCACAGTCACA	198
Gls	GGAGGGAAGGTTGCTGATTA	AGGACTGAAGACAAAAGGGAAC	133
Ucp2	GGTCGGAGATACCAGAGCAC	ATGAGGTTGGCTTTCAGGAG	173
Npc1	GCCGATTACCACACGCACTT	AACACAAGCCACACGGGAACAG	122

Table S2. Daily amounts of supplemented leptin and myo-inositol from day 1 to day 20 of lactation

**Day of lactation**	**Leptin (ng)** [1]	**Myo-inositol (mg)** [2]
1	1.0	0.43
2	2.0	0.85
3	3.0	1.28
4	4.0	1.70
5	5.0	2.13
6	6.3	2.67
7	7.5	3.20
8	8.8	3.73
9	10.0	4.26
19	11.3	4.80
11	15.6	5.33
12	17.2	5.86
13	18.8	6.39
14	20.3	6.93
15	21.9	7.46
16	23.5	7.99
17	25.0	8.52
18	26.6	9.06
19	39.4	9.59
20	43.8	10.12

[1] Picó, C.; Oliver, P.; Sánchez, J.; Miralles, O.; Caimari, A.; Priego, T.; Palou, A. The intake of physiological doses of leptin during lactation in rats prevents obesity in later life. *Int J Obes (Lond)*. **2007**, *31(8)*, 1199-209.

[2] Castillo, P.; Palou, M.; Otero, D.; Núñez, P.; Palou, A.; Picó, C. Sex-specific Effects of Myo-inositol Ingested During Lactation in the Improvement of Metabolic Health in Adult Rats. *Mol Nutr Food Res*. **2021**, *65(11)*, e2000965.

Figure S1. Percentage of NPY and αMSH positive neurons in the arcuate nucleus of the hypothalamus

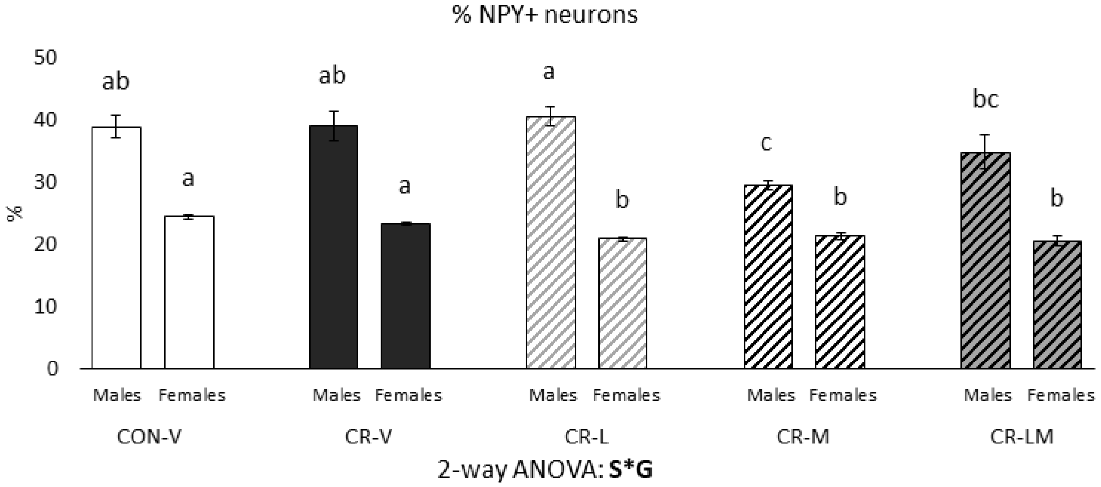

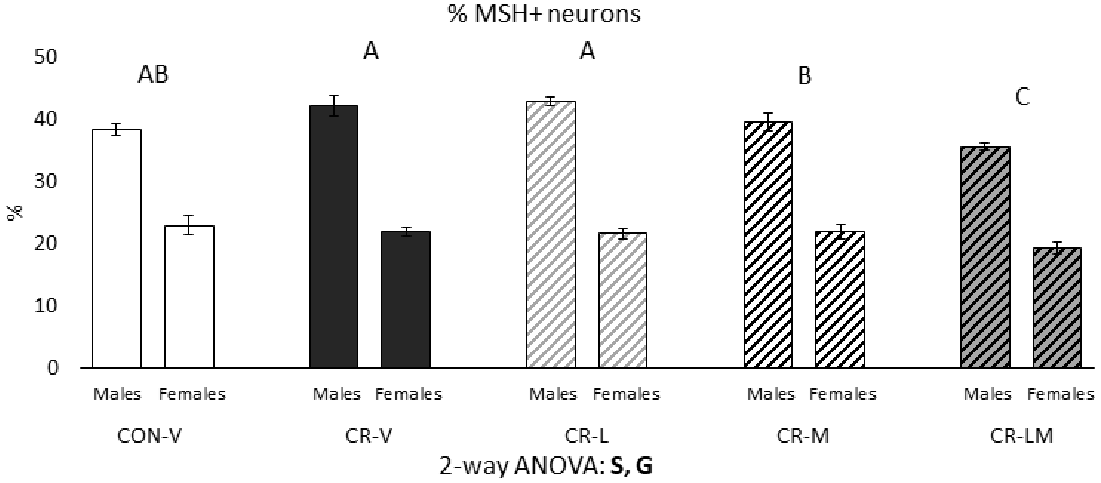

Percentage of NPY and αMSH positive (NPY+ and αMSH+) neurons in the arcuate nucleus of the hypothalamus of 25-day-old male and female offspring of control (CON) and 25% gestational calorie-restricted (CR) dams. Data are means ± s.e.m (n=4-6). Statistics: Two-way ANOVA was performed to assess the effects of sex and group (CON-V, CR-V, CR-L, CR-M and CR-LM). One-way ANOVA was carried out to determine differences between groups in each sex separately, when previous two-way ANOVA showed interactive effect between sex and group. ANOVA was followed by a least significant difference (LSD) post-hoc test. Symbols: S, effect of sex; G, effect of group; S*G, interactive effect between sex and group; A≠B≠C ($P<0.05$, two-way ANOVA); a≠b≠c ($P<0.05$, one-way ANOVA). Abbreviations: vehicle (V), leptin (L), myo-inositol (M), leptin and myo-inositol (LM).
